# The Association Between Physical Activity and Physical Literacy Among Adolescents: A Cross-Sectional Study

**DOI:** 10.3390/bs16081358

**Published:** 2026-08-07

**Authors:** Song Qian, Xuebin Wang, He Dan, Yuke Qin

**Affiliations:** 1 School of Marxism, Shanghai University of Sport, No. 399, Changhai Road, Yangpu District, Shanghai 200438, China; 2 Department of Physical Education, Shanghai Jiao Tong University, Shanghai 200240, China; 3 Post-Doctoral Station of Sport Science, Fujian Normal University, Cangshan Campus, No. 8 Shangsan Road, Cangshan District, Fuzhou 350007, China; 4 School of Journalism and Communication, Shanghai University of Sport, No. 399, Changhai Road, Yangpu District, Shanghai 200438, China; 5 School of Education, City University of Macau, Avenida Padre Tomás Pereira, Taipa, Macau 999078, China; m22092100147@cityu.mo

**Keywords:** physical literacy, physical activity, adolescents, moderate-to-vigorous PA, household PA, school-based PA

## Abstract

This study examined the association between physical activity (PA) patterns and physical literacy (PL) among adolescents in Shanghai, China. The researchers conducted a cross-sectional survey between December 2021 and February 2022. They recruited 1624 adolescents aged 11 to 18 from schools in various districts of Shanghai using stratified sampling. Out of these, 1500 provided complete and valid questionnaires for analysis. The researchers assessed PL using a validated adolescent Physical Literacy Questionnaire and measured PA with an updated PA questionnaire that included school-based, household, transportation, and leisure activities. The demographic factors considered included gender, school stage, academic performance, and family socioeconomic status (SES). Two-way multivariate analysis of variance (MANOVA) and multiple linear regression analyses were used to explore group differences and the connections between PA and PL. The MANOVA results showed significant differences in PA based on gender (F(1,1498) = 7.94, *p* < 0.001) and school stage (F(1,1498) = 9.79, *p* < 0.001). However, no significant differences were found concerning academic performance (F(1,1498) = 2.12, *p* = 0.081) or SES (F(1,1498) = 1.90, *p* = 0.072). On the other hand, PL varied significantly by school stage (F(1,1498) = 9.55, *p* < 0.001), academic performance (F(1,1498) = 8.84, *p* < 0.05), and SES (F(1,1498) = 7.94, *p* < 0.05), but not by gender (F(1,1498) = 1.90, *p* = 0.121). Furthermore, multiple regression analyses revealed that involvement in school-based, transportation, and leisure-time PA was positively associated with adolescents’ PL, whereas household PA was not. These findings suggest that adolescents’ participation in PA and their PL vary among different demographic groups. Additionally, different PA contexts are associated with varying degrees of PL development. Because of the cross-sectional nature of the study, these findings should be seen as associative rather than indicating a causal relationship. Programs that encourage participation in school-based, transportation, and leisure activities may help improve adolescents’ physical literacy.

## 1. Introduction

Physical literacy (PL) refers to the motivation, confidence, physical competence, knowledge, and understanding that enable individuals to maintain lifelong engagement in physical activity (PA) (Whitehead, 2010). It is widely recognized as a multidimensional construct that not only supports sustained PA participation but also contributes to broader physical, psychological, and social well-being across the lifespan. Recent theoretical and empirical models increasingly emphasize that PL is both an outcome and a developmental process shaped through engagement in diverse movement experiences, particularly moderate-to-vigorous PA, which has been shown to play a meaningful role in its development and associated health outcomes (Widiawati et al., 2025; Durden-Myers et al., 2026).

In recent years, research on PL has expanded rapidly, with a notable shift toward empirical validation, intervention studies, and integrative reviews linking PL to fitness, health behaviors, and psychosocial outcomes. Recent evidence demonstrates that structured school-based PA programs can significantly enhance fitness and psychosocial well-being in pre-adolescents, highlighting the importance of educational settings in shaping PL development (Vivek & Parasuraman, 2026). Similarly, systematic reviews and meta-analyses conducted in 2024 have confirmed consistent positive associations between PL and cardiorespiratory fitness in children and adolescents, reinforcing the importance of PL as a health-related construct (Jiang et al., 2025). Emerging international studies from 2022 to 2025 further suggest that PL is closely associated with PA levels and sedentary behavior patterns. However, the strength and direction of these relationships vary across populations and measurement approaches (Ying et al., 2025).

Recent empirical research has also increasingly focused on refining measurement frameworks and validating PL across diverse cultural contexts (Rajkovic Vuletic et al., 2026; Zhong et al., 2026). Studies conducted in South Asia and other regions have contributed to the adaptation and validation of assessment tools such as CAPL-2, demonstrating acceptable reliability and cross-cultural applicability (Hadier et al., 2023, 2025). Furthermore, longitudinal and cross-sectional investigations have explored the mediating role of physical fitness in the relationship between PA and PL, suggesting complex interdependencies rather than simple linear effects (Hadier et al., 2024). Normative and population-based studies have also been developed to establish reference standards for PL in children, reflecting the field’s growing maturity (Hadier et al., 2025). Together, these studies reflect a clear trend in the recent literature toward more diversified methodologies, cross-cultural validation, and integrated health frameworks.

Despite these advances, existing research still presents several limitations. First, many studies treat PA as a single, unified construct, without distinguishing among domains such as school-based, household, transportation, and leisure-time activities. Second, although growing evidence suggests that PL and PA are interrelated, findings remain inconsistent across age groups, cultural contexts, and measurement tools, indicating a lack of clarity regarding the strength and specificity of these associations (Ying et al., 2025; Zhong et al., 2026). Third, while both international and emerging studies have expanded in scope, fewer empirical investigations have systematically examined these relationships among primary and secondary school students in mainland China using large-scale datasets and differentiated activity domains.

It is also important to acknowledge that the relationship between PL and PA is likely bidirectional. While PL facilitates greater engagement in PA, PA participation contributes to the development of PL through experiential learning processes. However, most existing studies, including the present one, are cross-sectional and therefore cannot establish causal direction. Accordingly, the present study focuses on statistical associations rather than causal pathways.

Given the ongoing concern that PA levels and PL development among Chinese adolescents remain suboptimal, there is a clear need for more fine-grained empirical evidence that distinguishes between different types and intensities of PA. Such evidence is essential for informing school-based physical education practices and promoting more effective youth activity strategies. To address these gaps, this study adopts an empirical approach to examine how specific domains of PA relate to PL outcomes among adolescents in mainland China.

Based on the existing literature and the identified research gaps, the present study aimed to examine the associations between physical activity (PA) and physical literacy (PL) among adolescents in Shanghai, China. Specifically, the study sought to:

▪ Examine the association between overall PA participation and adolescents’ PL;
▪ Investigate whether different PA domains (school-based, household, transportation, and leisure-time activities) are associated with adolescents’ PL in different ways;
▪ Compare PA and PL across key demographic characteristics, including gender, school stage, academic achievement, and family socioeconomic status (SES).

## 2. Research Design

### 2.1. Research Object

According to the principle of stratified sampling, with urban and suburban areas as reference factors, 14 schools in Huangpu District, Yangpu District, Hongkou District, Putuo District, Pudong New Area, Songjiang District, Minhang District, Jing’an District, and Changning District of Shanghai were selected for the study. A total of 1624 junior and senior high school students aged 11–18 were selected for the survey. The questionnaires were distributed to respondents, collected, and processed; 1500 valid questionnaires were included after excluding invalid ones (incomplete or incorrect answers or obvious problems with answers), with an efficiency rate of 92.36%. After the study was approved by the research ethics committee at Shanghai University of Sport, written informed consent was obtained from all participants.

#### Participants and Sample Size

According to the principles of stratified random sampling, Shanghai was first stratified into urban and suburban districts to ensure adequate geographical representation. Fourteen junior and senior high schools were then randomly selected from nine districts, including Huangpu, Yangpu, Hongkou, Putuo, Pudong New Area, Songjiang, Minhang, Jing’an, and Changning. Eligible students aged 11–18 years were subsequently recruited from the selected schools.

The minimum required sample size was estimated using the standard formula for large population surveys:

N = Z^2^P(1 − P)/d^2^

where Z = 1.96 (95% confidence level), P = 0.50 (maximum variability), and d = 0.05 (margin of error). This calculation yielded a minimum required sample of 384 participants. Given the stratified sampling design, subgroup comparisons, multivariate analyses, and potential nonresponse, a substantially larger sample size was targeted to ensure adequate statistical precision and power.

A total of 1624 questionnaires were distributed to eligible students. After excluding incomplete questionnaires, questionnaires with inconsistent responses, and questionnaires containing obvious response errors, 1500 valid questionnaires were retained for analysis, representing a valid response rate of 92.36%. The final sample size considerably exceeded the minimum required, providing sufficient statistical power for the multivariate analyses conducted in this study.

The study was approved by the Research Ethics Committee of Shanghai University of Sport, and written informed consent was obtained from all participants and their legal guardians before data collection.

### 2.2. PL Measurement Questionnaire

Our method was based on the Perceived Physical Literacy Instrument (PPLI) developed by Rui-Si Ma et al. The original version of the PPLI was developed by Raymond Kim Wai Sum and other scholars to measure adolescents’ PL, and research has confirmed its high reliability and validity in assessing adolescents’ PL (Sum et al., 2018). The PPLI is a simplified version adapted by Rui-Si Ma for the actual situation in mainland China. The PPLI measures PL in three dimensions, namely self-confidence and motor ability, motivation, and environmental interaction, with eight questions. A 5-point Likert scale was used, with each item scored on a scale of 1–5 from “not at all” to “fully”, yielding a total score of 5–40. The items include: “I have the basic skills needed to participate in sports”, “my body is in a healthy state for my age”, and “I understand the health benefits of sports”. In this study, the PPLI was translated, back-translated, and corrected using a typical inter-translation procedure (the same method was used for the IPAQ-A questionnaire below). Two linguists proficient in English translated the English items; two physical education experts, also proficient in English, corrected and revised the translations; and two foreign teachers who had not seen the original version of the scale back-translated the translations into English. The above procedure was repeated several times until the semantics, expressions, and connotations of all the English and Chinese items matched. In this study, the questionnaire’s reliability was assessed using the retest method. A total of 400 students were selected to complete the questionnaire twice, with a 2-week interval between administrations. The consistency test between the two yielded a coefficient of 0.732, indicating good reliability. The internal consistency of the questionnaire was assessed using Cronbach’s alpha; the PL Questionnaire (PPLI) had an alpha of 0.888, indicating good internal consistency. The questionnaire’s validity in measuring perceived PL among Chinese adolescents was examined through exploratory (factor loadings ranged from 0.68 to 0.93) and confirmatory (factor loadings ranged from 0.60 to 0.92) factor analyses (Sum et al., 2018).

### 2.3. PA Measurement Questionnaire

PA was measured using the IPAQ-A (International PA Questionnaire-Adolescent), a questionnaire specific to adolescent PA. Studies have confirmed the high reliability and validity of the IPAQ-A to measure adolescents’ PA (Anna et al., 2021). The IPAQ-A covers four areas, namely school, household chores, transportation, and leisure activities, and it assesses participants’ PA levels by measuring walking, moderate-intensity, and high-intensity PA lasting at least 10 min over the past 7 days (5 school days and 2 weekend days). The consistency test of the IPAQ-A questionnaire, retested at 2-week intervals in this study, yielded a consistency coefficient of 0.654, indicating good retest reliability, and a Cronbach’s α of 0.764, indicating good internal consistency. Validity was assessed using Spearman’s rank correlation with the Caltrac accelerometer (*p* < 0.05, r = 0.50). The results showed that the long version of IPAQ-A had intraclass correlation coefficients above 0.7 for PA (Anna et al., 2021).

### 2.4. Measurement of Demographic Factors

Students’ gender, grade level, academic achievement, and family socioeconomic status were measured, with information on gender and grade level collected via questionnaires. The researchers collected academic achievement data by asking the class teacher for the class ranking in the last two midterms or final exams. The rankings were divided into three levels to classify students’ academic achievement as excellent, good, or poor. Family socioeconomic status (SES) was determined from the questionnaire based on students’ parents’ education level, parents’ occupation, and monthly family income. These six variables were assigned different weights, with the classification and assignment borrowed from Yin et al. (2020) and N. Zhang et al. (2013), and the weighted scores were saved as standard scores, using the standard scores of parents’ education level, parents’ occupation, and monthly family income. The family socioeconomic index was computed after saving the factor scores, and the scores for the whole sample were then divided into three levels: high, medium, and low.

### 2.5. Statistical Analysis

The questionnaire results were statistically analyzed using SPSS 19.0. Descriptive statistics helped us examine the current levels of youth physical literacy (PL) and physical activity (PA). We used independent-samples t-tests to assess demographic differences between PL and PA. We also conducted multiple linear regression analyses to explore the connections between various types and intensities of PA and PL. We set the significance level at *p* < 0.05.

## 3. Research Results

### 3.1. Current Characteristics of Adolescents’ PL and PA

(1) Adolescents developed appropriate PL. The overall mean score of adolescents’ PL was 31.73, with scores of 12.33, 11.87, and 7.54 for the three dimensions of motivation, confidence and athletic ability, and environmental interaction, respectively, i.e., the highest score for motivation, the second highest for confidence and athletic ability, and the lowest for environmental interaction. The mean scores for each PL dimension among Chinese adolescents are not significantly different from those reported by Cheng Zhang (12.1, 11.3, and 7.4 for the three dimensions, respectively) (C. Zhang et al., 2022) and Hagströmer et al. (2008) (11.92, 11.09, and 7.27 for the three dimensions, respectively), and the distribution patterns of scores are consistent, indicating that Chinese adolescents are strongly motivated to play sports and have certain athletic confidence and athletic ability, but their communication skills with others are weak. Years of physical education course learning experience, the National Student Physical Fitness Standard test, and multi-channel physical culture propaganda for the adolescents who participated in this study have taught them general sports skills and basic fitness knowledge, so they have some knowledge of the benefits of exercise, such as physical fitness enhancement, stress relief, and physical and mental pleasure, and also have the confidence and motivation to participate in physical exercise. The lower score on the interactive aspect of PL might relate to various contextual factors. One possible reason is that communication norms in traditional Chinese culture emphasize harmony, modesty, and indirectness. These norms could affect students’ willingness to participate in classroom discussions and express themselves openly. However, since this study did not directly examine cultural values or communication practices, this interpretation should be seen as tentative rather than definitive and requires further research (Hadier et al., 2023; Ying et al., 2025).

(2) The phenomenon of “non-parity” in PA among adolescents is prominent. The survey showed that the average total PA of adolescents was 63.52 MET/h/wk, which was significantly lower than that of Hong Kong adolescents (69.55 MET/h/wk) and European adolescents (71.89 MET/h/wk) (Hadier et al., 2024) (See Table 1). PA in school (all abbreviated as “school” in the table) was 34.89 MET/h/wk, PA in household activities (all abbreviated as “household” in the table) was 4.91 MET/h/wk, and PA in transportation (all abbreviated as “transportation” in the table) was 6.68 MET/h/wk. This shows that school remains the primary setting for PA and exercise. The combined total of household chores, transportation trips, and leisure activities did not exceed the total of school activities. This also reflects that the extracurricular activities of primary and secondary school students are relatively insufficient. In addition, as family economic conditions improve, youth participation in leisure activities is increasing, ranking second in this survey. Recreational sports activities have many positive effects on adolescent development; for example, mountaineering activities have provided significant physical and psychological benefits, improving physical mobility and promoting cardiorespiratory fitness, while improving mood, reducing anxiety and restlessness, and fostering self-identity and self-satisfaction (Geng et al., 2023). Therefore, it is recommended that various measures be taken to encourage youth to increase their participation in recreational sports activities outside of school hours.

### 3.2. Demographic Differences in Adolescents’ PL and PA

To examine whether there are demographic differences in adolescents’ PL and PA, the following two-way multivariate analysis of variance (MANOVA) tests were conducted with gender, school level, academic achievement, and family socioeconomic status as independent variables, and PA and PL scores as dependent variables, respectively, and the results are shown in Table 2.

The multivariate analysis showed significant gender differences in adolescents’ physical activity (PA). Boys reported much higher PA levels than girls (F(1,1498) = 7.94, *p* < 0.001). Boys also had slightly higher physical literacy (PL) scores, but this difference was not statistically significant. These findings support previous studies (Tremblay et al., 2018). Contemporary expectancy-value theory suggests that gender-role stereotypes affect behavior through self-perceptions. Girls are often seen as quieter and less active than boys, which may cause them to feel conflict when participating in sports. These perceptions can lower girls’ enjoyment of physical activity and discourage them from participating, leading to lower PA levels. In contrast, physical education curricula provide similar opportunities for both boys and girls to learn sport-related skills, making gender differences in PL less noticeable.

Significant differences were also found between school stages. Junior high school students showed significantly higher PA (F(1,1498) = 9.787, *p* < 0.001) and PL (F(1,1498) = 14.638, *p* < 0.001) than senior high school students. These findings match previous research. One reason might be the growing academic pressure as students move to higher grades. Junior high students usually have more free time and chances to be active. In contrast, senior high students spend much more time on homework and academic tutoring, which reduces their opportunities for exercise (Dong, 2017). Moreover, the physical education entrance exam policy has strengthened schools’ focus on physical education by improving curriculum implementation and encouraging participation in school and extracurricular sports. These activities help students develop motor skills and sport-related knowledge, leading to higher PL.

No significant differences in PA were noted among academic achievement groups. However, students with excellent (F(1,1498) = 8.62, *p* = 0.006) and good (F(1,1498) = 9.35, *p* = 0.004) academic performance had significantly higher PL than those with poor academic achievement. While earlier studies reported positive links between PA and academic success (Darling, 2005), self-reported PA did not show a significant connection to academic performance in this study. This difference might stem from the gap between objective and self-reported measures of PA, as objective assessments often show stronger links with academic results. It could also be due to confounding factors or limitations in observational research (Fang, 2020). Therefore, future studies should use reliable, objective measures of PA to better understand its role in academic success and how different types of physical activity interact (Jaakkola et al., 2015). In contrast, academic achievement showed a clear positive connection with adolescents’ PL. Knowledge and understanding are key parts of PL, as they help individuals know what to do, how to do it, and when to use movement skills effectively (Edwards et al., 2017; Ennis, 2015; Whitehead, 2019a, 2019b; Cale & Harris, 2018). Students with stronger academic skills may learn and apply sport-related knowledge more effectively, contributing to higher levels of PL.

No significant differences in PA were found among family socioeconomic status (SES) groups. However, from the post hoc results, adolescents from middle-SES (F(1,1498) = 9.14, *p* = 0.004) and high-SES (F(1,1498) = 6.95, *p* = 0.004) families had significantly higher PL than those from low-SES families. This suggests that family SES relates to adolescents’ PL but not to their PA participation. The lack of significant SES differences in PA aligns with previous research (Barr-Anderson et al., 2017). One possible reason is the growth of public sports facilities and community physical activity options through national programs such as the National Fitness Plan (2021–2025), which has improved access to sports and encouraged participation across socioeconomic groups (Zhu & Liang, 2021). However, families with more financial resources are more likely to provide organized recreational sports, transport to activities, and financial support for extracurricular involvement. These experiences help develop movement skills, confidence, motivation, and social interaction, fostering higher PL levels (Chamberlain, 1983; Hong & Li, 2023). Interviews with participants also showed that adolescents from lower-income families participated less often in recreational sports, limiting their chances to develop the cognitive, physical, and social skills that support physical literacy.

### 3.3. Regression Analysis of Teenagers’ PL and PA

#### 3.3.1. Regression Relationship Between Different Types of Physical Activities and PL

The total PL score (Model I), self-confidence and motor ability (Model II), motivation (Model III), and environmental interaction (Model IV) were used as dependent variables, and PA in school, household activities, transportation trips, and leisure activities were used as independent variables. Gender and grade were used as control variables (previous results showed significant differences in PA by gender and age), and multiple linear regressions were conducted. The results are shown in Table 3.

The results of the regression analysis (Table 3) showed that the following: (i) In Model I, school, household activities, and leisure activities had a significant effect on the total PL score, but transportation trips were not significant. Based on the magnitudes of the regression coefficients, the total PL score was influenced by household activities (0.064), school (0.021), and leisure-time PA (0.014), in descending order. (ii) In Model II, school, household activities, and leisure activities had significant effects on self-confidence and motor ability, but transportation trips were not significant. The effects were in descending order of household activities (0.019), school (0.010), and leisure activities (0.006). (iii) In model three, school, household activities, and leisure activities have a significant effect on motivation. However, transportation trips were not significant, and their influence was in descending order: household activities (0.023), school (0.007), and leisure activities (0.005). In Model IV, household activities (0.022) had a significant effect on environmental interaction, whereas school, transportation trips, and leisure activities did not.

The results of the combined regression analyses show that household activities have a strong impact on overall PL and its various dimensions, with the highest regression coefficients found among the activity domains examined. This suggests that household activities may be crucial for the development of PL in adolescents. Previous studies have indicated that self-care and household tasks can significantly improve fine motor coordination in school-aged children (Huang et al., 2026). For instance, tidying rooms, organizing personal belongings, and using household tools can enhance hand–eye coordination, body control, and movement coordination (Hu et al., 2022). Better motor coordination can help children learn movement skills, participate in physical activities, and boost their self-confidence (Song et al., 2022). Additionally, engaging in household tasks allows adolescents to practice self-observation, self-evaluation, and taking responsibility, which may help build their self-esteem, self-confidence, and positive self-regulation (Y. Zhang & Mustafa, 2023). However, the stronger effect of household activities compared to school- and leisure-time physical activities warrants careful consideration. Rather than being the only factor boosting PL, household activities may also reflect broader family and social influences. For example, adolescents who take on more household tasks may come from families with higher parental involvement, better communication, greater emphasis on responsibility, and more opportunities for experiential learning. These family traits have been linked to supporting children’s physical, psychological, and social growth, which are key parts of PL. Also, differences in adolescents’ participation in household activities may reflect variations in family socioeconomic status, family structure, or cultural expectations regarding children’s responsibilities, which could indirectly affect PL outcomes.

Therefore, the link between household activities and PL should not be taken as proof that household tasks alone lead to better PL. Instead, these activities may provide crucial context for adolescents to develop movement skills, self-confidence, motivation, and social interaction abilities while benefiting from supportive family environments. Future research should include factors such as parental involvement, family income, parenting styles, and family communication patterns to better understand the direct and indirect links between household activities and PL development.

It is also important to note that household activities were the only activity domain significantly linked with environmental interaction. During these tasks, parents and adolescents often talk, share experiences, discuss responsibilities, and give feedback, creating valuable chances for social learning and personal growth. Such interactions can improve adolescents’ communication skills and their ability to engage with their surroundings. Since the environmental interaction aspect of PL focuses on communication and social participation, these family interactions may help explain the unique role of household activities in this area. As a result, both families and schools should consider how the communicative and collaborative elements of household activities can support adolescents’ overall PL development.

It is also important to highlight that household activities were the only domain significantly linked to environmental interaction. During these tasks, parents and adolescents often communicate, share experiences, discuss responsibilities, and give feedback. These interactions create valuable opportunities for social learning and personal growth. Such exchanges enhance adolescents’ communication skills and their ability to engage effectively with their surroundings. Since the environmental interaction aspect of PL highlights communication and social involvement, these family-based interactions may help explain why household activities are uniquely significant in this area. Therefore, both families and schools should consider how the communicative and collaborative nature of household activities can be leveraged to support adolescents’ overall PL development.

PA in school had a significant effect on PL, self-confidence, motor ability, and motivation, with the second-highest regression coefficients, indicating that it also significantly impacts students’ PL development. Physical education is an ideal setting to develop PL by offering a wide variety of exercise content in structured or unstructured formats, guided by relevant standards and practice recommendations that help children reach their full potential as learners and thus contribute to the development of PL (Castelli & Ward, 2012). Given the long school hours of Chinese youth and the fact that schools are the main settings for most PA, it is easy to see the positive effect of school PA on youth perceptions of PL. However, although adolescents perform the most total PA at school, they rank only second in terms of their influence on adolescents’ perceptions of PL. There are two main reasons for this phenomenon: As a new concept, physical education teachers may not yet have the ability or time to teach students PL content in a short period of time. Research on PL in China began in 2016, and the first PL assessment system was introduced in 2019. In general, research on PL in China is still in its initial stage. The lag in theoretical research impedes the development of practical work, and it is difficult for physical education teachers to teach students because they do not fully understand the meaning and structure of PL. ② No curriculum implementation plan has been designed and developed with PL as the core, which adversely affects school physical activities aimed at promoting PL among youth. Western countries have actively supported the growth of youth physical literacy (PL) through various school-based physical activity (PA) programs and national policies. Notable examples include *Physical Literacy in the United States: A Model, Strategic Plan, and Call to Action in the United States* and *Getting Australia Moving: Establishing a Physically Literate and Active Nation in Australia*. These initiatives have made PL a key part of lifelong health, participation in physical activity, and educational development.

In China, the focus on PL has also increased in recent years. In 2020, the Chinese government released *Opinions on Comprehensively Strengthening and Improving School Sports in the New Era*. This document clearly pointed out the need to develop physical literacy among children and adolescents. However, even with this policy support, the document only offers broad strategic advice. It does not provide clear steps for implementation, teaching methods, or assessment criteria to encourage PL development in schools effectively. Therefore, in order to develop the positive role of school physical activities in the cultivation of youth PL to the greatest extent, government departments should work on both teachers and curriculum construction; for teachers, they need to accelerate the deepening of physical education teachers’ knowledge of PL, and for curriculum construction, they need to accelerate the revision of PL-oriented curriculum standards and fully integrate PL into physical education curriculum standards (Yin et al., 2020) to ensure PL development in school practice.

Leisure activities had significant effects on the dimensions of PL as a whole, self-confidence, motor ability, and motivation, and the regression coefficients ranked third, indicating that the development of youth PL cannot be separated from the support of leisure activities. As a result of the school curriculum and teaching content restrictions, most youth leisure activities are chosen based on personal interest and more positive attitudes, and in the process of participation, require the courage to undertake and solve the difficulties faced. In the long run, youth gain self-confidence and develop a good habit of long-term participation, which has a positive impact on the development of youth PL. As Whitehead stated, self-directed selection of PA can be effective in improving PL (Whitehead, 2019b). The positive effect of leisure activities on PL has been demonstrated in other studies. However, the present study differs from other studies in that the influence of leisure activities on youth PL development did not exceed that of domestic and school activities. The reason may be that the total number of adolescent students involved in leisure PA in this study remains insufficient. Molina et al.’s study showed that only very high levels of leisure activity (>3000 MET/min/wk) improved self-esteem among college students (Molina-García et al., 2011), and an increase in self-esteem leads to a corresponding increase in motivation for exercise (Xu et al., 2020). Related studies have also shown that creating a curriculum environment with a clear division of responsibilities and frequent communication and interaction can be effective in achieving PL development (Choi et al., 2021). However, adolescents’ program choices for leisure activities likely do not share this characteristic; for example, surveys have shown that walking, swimming, and badminton are the top three choices of leisure physical activities among young and middle-aged adults (Liu et al., 2021). The preference for single-player programs may also be a reason recreational sports have not had a greater impact on PL development. In order to deeply exploit the efficacy of leisure activities in the development of youth PL, the following aspects can be addressed: for individuals, the total number of leisure sports activities needs to be further increased; for families, the accompaniment of youth at leisure sports activities needs to be enhanced, and positive emotions associated with leisure activities need to be increased.

#### 3.3.2. Regression Relationship Between PA of Different Intensities and PL

Multiple linear regressions were conducted with total PL score (Model I), self-confidence and athletic ability (Model II), motivation (Model III), and environmental interaction (Model IV) as dependent variables; walking, moderate intensity, and high intensity PA as independent variables; and grade level, academic achievement, and family socioeconomic status as control variables (the previous results showed significant differences in these entries corresponding to PL scores), and the results are shown in the following table.

The results of the regression analysis (Table 4) showed the following: (i) In Model I, moderate- and high-intensity PA significantly affected the total PL score, whereas walking did not. The impact decreased from moderate-intensity activity (0.036) to high-intensity activity (0.019). (ii) In Model II, both moderate-intensity and high-intensity PA significantly influenced self-confidence and motor ability, whereas walking remained non-significant. Moderate-intensity PA (0.011) had a greater impact than high-intensity PA (0.009). (iii) In Model III, moderate-intensity and high-intensity physical activities significantly affected motivation, with walking showing no significant effect. The effects were stronger for moderate-intensity activity (0.013) compared to high-intensity activity (0.006). (iv) In Model IV, only moderate-intensity PA (0.012) significantly impacted environmental interaction, while walking and high-intensity PA did not have significant effects. Previous research has shown that children and adolescents who meet the Canadian guideline of at least 60 min of moderate-to-vigorous PA each day demonstrate significantly higher levels of physical skills, motivation, and confidence than those who do not (Belanger et al., 2018). The findings of this study support this evidence and further highlight the role of participation in moderate-to-vigorous PA in the development of PL. Positive experiences from PA can improve enjoyment, self-efficacy, and motivation, encouraging ongoing engagement in active lifestyles. Regular participation in PA also provides chances for social interaction with peers, teachers, and family members, leading to stronger relationships and a greater sense of belonging.

Meanwhile, some studies suggest that high-intensity PA might be more beneficial than moderate-intensity activity for improving PL. However, the findings of this study do not completely support that idea. Differences in participant characteristics may partly explain this inconsistency, as earlier research often focused on university students rather than adolescents in primary and secondary schools. Additionally, this study showed that adolescents engaged in substantially more high-intensity than moderate-intensity activity. Excessive participation in vigorous exercise can lead to fatigue, muscle soreness, and an increased risk of injury, thereby encouraging sedentary recovery behaviors and reducing the overall benefits of PA (Xiao et al., 2018). Therefore, moderate-intensity activity may offer a more sustainable and suitable way to improve PL among adolescents. It is noteworthy that moderate-intensity PA had a stronger effect than high-intensity activity across all aspects of PL. Besides boosting physical skills and confidence, moderate-intensity activities often foster communication, teamwork, and social interaction. This may explain why only moderate-intensity activity was significantly linked to environmental interaction. High-intensity activities usually require more physical effort and focus, which can limit opportunities for peer communication and collaboration.

In recent years, China has introduced several policy initiatives, like Opinions on Strengthening School Sports for the Overall Development of Students’ Physical and Mental Health and the Healthy China 2030 Planning Outline, to boost youth PA participation. Still, physical inactivity remains a major issue. The 2018 Chinese Children and Youth PA Report Card revealed that only 13.1% of young people met the recommended 60 min of daily moderate-to-vigorous PA, with participation rates dropping from primary to secondary school (Oh et al., 2019). This trend underscores the urgent need for effective strategies to promote both PA participation and PL development. Research on the dose–response relationship between PA and health has consistently shown that moderate-to-vigorous PA offers more health benefits than low-intensity activity (Lin & Xie, 2011). Beyond health promotion, recent studies increasingly recognize PL as a key factor for lifelong health and well-being in children and adolescents. A detailed bibliometric review indicates that PL is widely regarded as a crucial concept linking PA, health outcomes, and overall youth development (Urbano-Mairena et al., 2023). Similarly, a systematic review of positive youth development through sport and physical education highlights that meaningful engagement in PA supports not just physical competence but also confidence, social connections, and personal growth (Almeida et al., 2025). These findings align with current views of PL as a multidimensional construct encompassing physical skills, motivation, confidence, knowledge, and social engagement (Santos et al., 2022). From this perspective, moderate-intensity PA may strike a good balance between physical challenge and social interaction, aiding development in various areas of PL. Moreover, evidence indicates that PL is an important predictor of PA participation, revealing a two-way relationship in which physically literate adolescents are more likely to lead active lifestyles, and participation in PA helps strengthen PL (Öztürk et al., 2023).

The practical importance of this relationship is evident in intervention studies showing that programs focused on PL can effectively increase PA participation and improve health-related outcomes in adolescents (Nezondet et al., 2023). Recent meta-analytic evidence also indicates that structured PA interventions, including resistance training programs, positively affect various components of PL, especially physical competence, confidence, and motivation (Valle-Muñoz et al., 2025). These findings suggest that schools should focus not just on increasing the volume of activity but also on the quality and appropriateness of PA experiences.

Further support for the value of PL comes from evidence that children with higher levels of PL are more likely to meet recommended PA guidelines, even in challenging situations such as the COVID-19 pandemic (Mazzoli et al., 2026). This highlights the importance of developing PL to encourage long-term engagement in PA, rather than aiming only for short-term behavior changes. The findings also add to the growing body of research highlighting PL as an ongoing developmental journey. Recent studies have shown how PL experiences in adolescence shape participation patterns and health trajectories later in life (Carl et al., 2026; Cowan & Pushkarenko, 2026). Thus, promoting moderate- and high-intensity PA in adolescence may yield benefits that extend beyond immediate physical health outcomes, supporting lifelong active living. Given these findings, future educational and sports policies should place more importance on integrating PL into school-based PA programs. Recent research on innovative approaches to assessing PL suggests that comprehensive monitoring systems can help educators evaluate students’ development and tailor interventions accordingly (Hogan et al., 2026). Therefore, schools, as key environments for adolescent PA, should create more opportunities for moderate-intensity PA while also adopting teaching and assessment practices that focus on PL. These efforts may offer a sustainable path to improving both PA participation and overall PL among young people.

## 4. Conclusions

This study examined the associations between PA and PL among adolescents in Shanghai. It involved a sample of 1500 valid participants. The results shed light on how various types and intensities of PA relate to adolescents’ PL. Overall, adolescents showed a moderate level of PL. They scored higher in motivation and confidence/physical competence and lower in environmental interaction. Participation in different activity types varied, with school-based PA representing the largest share of overall activity. Significant differences in PL were observed across school stage, academic achievement, and family socioeconomic status. However, gender differences were not statistically significant. In contrast, PA levels differed significantly by gender and school stage, but not by academic achievement or family socioeconomic status. These results imply that demographic and contextual factors may affect PL and PA differently among adolescents.

The regression analyses showed that physical activity at home, at school, and during leisure time was positively associated with PL outcomes. On the other hand, transportation-related PA did not show a significant link. It is important to interpret these findings carefully. Although household activities had larger regression coefficients, comparisons of their importance should be approached with caution. Coefficient size may be affected by differences in how measurements are taken and how activities are distributed. Thus, the results suggest that various PA types are associated with PL but do not establish a clear ranking of their influence. As for activity intensity, both moderate and vigorous physical activities are positively associated with PL. In contrast, walking did not show a significant connection to PL outcomes. Moderate-intensity activities had slightly stronger links across several dimensions of PL. However, these differences were usually small and do not mean that moderate activity is better than vigorous activity. Instead, the findings suggest that participating in moderate-to-vigorous PA may be connected to higher levels of PL among adolescents.

It is crucial to note that the cross-sectional design of this study limits causal conclusions. The relationships observed should be viewed as associations rather than as proof that specific types or intensities of PA directly improve PL. It is also possible that adolescents with higher levels of PL are more inclined to engage in PA, or that both are affected by other individual, family, or environmental factors. Furthermore, the use of self-reported measures, the focus on a single city, such as Shanghai, and the relatively modest explanatory power of the regression models should be considered when evaluating the findings. Despite these limitations, the study adds to the growing evidence that supports a relationship between PA and PL during adolescence. The findings indicate that schools, families, and community organizations can play important roles in encouraging adolescents’ participation in PA and their development of PL. Future longitudinal or intervention-based research is needed to clarify the direction of these relationships and identify which activity experiences are most beneficial for promoting PL among diverse adolescent populations.

## 5. Limitations and Future Research Directions

Several limitations should be kept in mind when interpreting the findings of this study. First, PA was measured using the self-reported International PA Questionnaire for Adolescents (IPAQ-A). While this tool has shown acceptable reliability and validity and is commonly used with adolescents, self-reported measures often suffer from recall bias, reporting inaccuracies, and the influence of social expectations. Adolescents may struggle to accurately recall the frequency, duration, and intensity of physical activities they engaged in during the past week. This could affect the accuracy of the estimates. Future research could address this limitation by using objective assessment tools, such as accelerometers, pedometers, wearable activity trackers, or direct observation methods. These could provide more precise measurements of PA behaviors and their relationships with PL.

Second, academic achievement was divided into three levels: excellent, good, and poor. This categorization was based on students’ rankings in their most recent midterm or final exams. Although this method served as a practical indicator of academic performance across a large sample, it does not fully account for variations in exam difficulty, grading practices, curriculum rigor, and academic standards across schools. Therefore, students in the same achievement category may not show similar levels of academic success. Future studies should consider using standardized academic assessments, district-wide exams, or nationally comparable achievement measures to make the performance indicators more consistent and comparable across different educational settings. Moreover, the study used a cross-sectional design, limiting our ability to draw causal conclusions about the relationship between PA and PL. While significant associations were found, we cannot definitively determine the direction of these relationships. Future longitudinal and intervention-based studies are needed to explore how different types of PA contribute to the development of PL over time and to identify the causal mechanisms behind these associations. Despite these limitations, the study provides valuable evidence about the current state of PL among Chinese primary and secondary school students. It also emphasizes the important role PA plays in supporting PL development. Addressing the noted methodological limitations will further enhance future research and deepen our understanding of the factors influencing PL among adolescents.

## Figures and Tables

**Table 1:** Summary of descriptive statistics of PL and PA among adolescents.

Physical literacy		Self-confidence and athletic ability	11.87
		Motivation	12.33
		Environmental interaction	7.54
		Total	31.73
PA (MET/h/wk)	By type	School	34.89
		Household	4.91
		Transportation	6.68
		Leisure activities	17.04
	By intensity	Walking	18.55
		Medium intensity	16.23
		High intensity	28.74
		Total	63.52

**Table 2:** List of variance analysis of adolescents’ PL and PA.

Variables		*n*	Physical Activity			Physical Literacy		
			M	SD	*p*	M	SD	*p*
**Gender**	**Male**	758	68.10	0.101	<0.001	31.98	0.040	0.121
	**Female**	742	58.84	0.103		31.48	0.040	
**Academic segments**	**Junior High School**	879	73.09	0.197	<0.001	32.36	0.099	<0.001
	**High School**	621	51.62	0.278		30.86	−0.141	
**Academic Performance**	**Excellent**	509	64.27	0.006	0.081	32.36	0.099	0.004
	**Good**	504	64.76	0.013		30.86	−0.141	
	**Poor**	487	63.54	0.021		32.36	0.099	
**SES**	**High**	500	64.95	0.016	0.072	32.84	0.178	0.026
	**Medium**	500	63.68	0.012		31.62	0.018	
	**Low**	500	64.02	0.004		30.74	−0.160	

**Table 3:** List of parameters of results obtained from multiple linear regression of PA and PL (N = 1500).

	**Model I (Total PL Score): *R* = 0.227, *R-Squared* = 0.052, *p* < 0.01**					**Model II (Self-Confidence and Motor Ability): *R* = 0.199, *R-Squared* = 0.036, *p* < 0.01**				
	**Regression Coefficient**	**Standard Error**	**Standard Regression Coefficient**	***t***	***p***	**Regression Coefficient**	**Standard Error**	**Standard Regression Coefficient**	***t***	***p***
Constants	32.096	0.927		34.606	<0.01	12.154	0.426		28.509	<0.01
School	0.021	0.006	0.097	3.491	<0.01	0.010	0.003	0.099	3.539	<0.01
Household	0.064	0.019	0.096	3.318	0.001	0.019	0.009	0.064	2.190	0.029
Transportation	−0.004	0.015	−0.007	−0.257	0.797	−0.008	0.007	−0.033	−1.126	0.260
Leisure activities	0.014	0.006	0.073	2.333	0.020	0.006	0.003	0.068	2.145	0.032
	**Model III (Motivation) *R* = 0.192, *R-Squared* = 0.0.37, *p* < 0.01**					**Model IV (Environmental Interaction): *R* = 0.193, *R-Squared* = 0.0037, *p* < 0.01**				
	**Regression Coefficient**	**Standard Error**	**Standard Regression Coefficient**	***t***	***p***	**Regression Coefficient**	**Standard Error**	**Standard Regression Coefficient**	***t***	***p***
Constants	12.227	0.378		32.354	<0.01	7.753	0.313		24.783	<0.01
School	0.007	0.002	0.083	2.966	0.003	0.004	0.002	0.051	1.813	0.070
Household	0.023	0.008	0.087	2.965	0.003	0.022	0.007	0.100	3.437	0.001
Transportation	−0.001	0.006	−0.004	−0.139	0.889	0.005	0.005	0.029	0.98	0.327
Leisure activities	0.005	0.002	0.066	2.076	0.038	0.003	0.002	0.046	1.457	0.145

**Table 4:** List of parameters of results obtained from multiple linear regression of PA and PL (N = 1500).

	**Model I (Total PL Score): *R* = 0.221, *R-Squared* = 0.049, *p* < 0.01**					**Model II (Self-Confidence and Motor Ability): *R* = 0.191, *R-Squared* = 0.036, *p* < 0.01**				
	**Regression Coefficient**	**Standard Error**	**Standard Regression Coefficient**	***t***	***p***	**Regression Coefficient**	**Standard Error**	**Standard Regression Coefficient**	***t***	***p***
Constants	32.285	0.918		35.169	<0.001	12.253	0.422		29.039	<0.001
Walking	0.007	0.010	0.022	0.711	0.477	0.001	0.004	0.006	0.203	0.839
Medium intensity	0.036	0.011	0.101	3.121	0.002	0.011	0.005	0.065	2.008	0.045
High Strength	0.019	0.006	0.098	2.983	0.003	0.009	0.003	0.102	3.074	0.002
	**Model III (Motivation): *R* = 0.184, *R-Squared* = 0.034, *p* < 0.01**					**Model IV (Environmental Interaction): *R* = 0.193, *R-Squared* = 0.0037, *p* < 0.01**				
	**Regression Coefficient**	**Standard Error**	**Standard Regression Coefficient**	***t***	***p***	**Regression Coefficient**	**Standard Error**	**Standard Regression Coefficient**	***t***	***p***
Constants	12.302	0.374		32.894	<0.001	7.766	0.309		25.094	<0.001
Walking	0.004	0.004	0.028	0.934	0.350	0.003	0.003	0.025	0.836	0.403
Medium intensity	0.013	0.005	0.093	2.863	0.004	0.012	0.004	0.105	3.221	0.001
High Strength	0.006	0.003	0.075	2.264	0.024	0.004	0.002	0.056	1.679	0.093

## Data Availability

The raw data supporting the conclusions of this article will be made available by the authors on request.
